# Distinguishing Type 2 Diabetes from Type 1 Diabetes in African American and Hispanic American Pediatric Patients

**DOI:** 10.1371/journal.pone.0032773

**Published:** 2012-03-07

**Authors:** Nancy Keller, Suruchi Bhatia, Jeanah N. Braden, Ginny Gildengorin, Jameel Johnson, Rachel Yedlin, Teresa Tseng, Jacquelyn Knapp, Nicole Glaser, Paula Jossan, Shawn Teran, Erinn T. Rhodes, Janelle A. Noble

**Affiliations:** 1 Children's Hospital and Research Center Oakland, Oakland, California, United States of America; 2 Division of Endocrinology and Diabetes, Sutter Pacific Medical Center, San Francisco, California, United States of America; 3 Michael E. Debakey Veterans Affairs Medical Center, Houston, Texas, United States of America; 4 Children's Medical Center, University of Texas Southwestern, Dallas, Texas, United States of America; 5 Department of Pediatrics, Pediatric Endocrinology, UC Davis Medical Center, Sacramento, California, United States of America; 6 Division of Endocrinology, Children's Hospital Boston, Boston, Massachusetts, United States of America; Universita Magna-Graecia di Catanzaro, Italy

## Abstract

**Objective:**

To test the hypothesis that clinical observations made at patient presentation can distinguish type 2 diabetes (T2D) from type 1 diabetes (T1D) in pediatric patients aged 2 to 18.

**Subjects and Methods:**

Medical records of 227 African American and 112 Hispanic American pediatric patients diagnosed as T1D or T2D were examined to compare parameters in the two diseases. Age at presentation, BMI z-score, and gender were the variables used in logistic regression analysis to create models for T2D prediction.

**Results:**

The regression-based model created from African American data had a sensitivity of 92% and a specificity of 89%; testing of a replication cohort showed 91% sensitivity and 93% specificity. A model based on the Hispanic American data showed 92% sensitivity and 90% specificity. **Similarities between African American and Hispanic American patients include**: (1) age at onset for both T1D and T2D decreased from the 1980s to the 2000s; (2) risk of T2D increased markedly with obesity. **Racial/ethnic-specific observations included**: (1) in African American patients, the proportion of females was significantly higher than that of males for T2D compared to T1D (p<0.0001); (2) in Hispanic Americans, the level of glycated hemoglobin (HbA1c) was significantly higher in T1D than in T2D (p<0.002) at presentation; (3) the strongest contributor to T2D risk was female gender in African Americans, while the strongest contributor to T2D risk was BMI z-score in Hispanic Americans.

**Conclusions:**

Distinction of T2D from T1D at patient presentation was possible with good sensitivity and specificity using only three easily-assessed variables: age, gender, and BMI z-score. In African American pediatric diabetes patients, gender was the strongest predictor of T2D, while in Hispanic patients, BMI z-score was the strongest predictor. This suggests that race/ethnic specific models may be useful to optimize distinction of T1D from T2D at presentation.

## Introduction

Pediatric type 1 diabetes (T1D) and type 2 diabetes (T2D) patients have become more similar to each other than they were 25 years ago. T1D patients are increasingly apt to be overweight/obese, and the age of T2D diagnosis is decreasing to the point that a patient as young as eight years old may no longer simply be assumed to have T1D, rather than T2D [1], [2], [3], [4], [5]. The rising incidence of T2D is particularly striking in the African American (AA) and Hispanic American (HA) pediatric populations, who are particularly at risk for T2D due to genetic and environmental influences [6], [7], [8]. African American and Hispanic American children from low income families are at high risk for T2D and are often underinsured or have no medical insurance with limited access to medical treatment [9], [10].

In this study, data from African American and Hispanic American pediatric diabetes patients were obtained retrospectively to examine differences between T1D and T2D patients at onset and to determine whether or not those differences could be utilized to create a simple, rapid, and inexpensive test to aid in the diagnosis of diabetes type in these populations. Such a test would help with appropriate allocation of scarce resources, such as diabetes educator time. In addition, this test is intended to help with and lead to prompt initiation of medical management with appropriate family education and care plans.

The study reported here was made to determine: (1) whether or not clinical observational data recorded at presentation could be used to predict T2D and (2) whether or not measurable and significant racial/ethnic differences in clinical observations might necessitate race/ethnic specific diagnostic aids to differentiate between T1D and T2D at presentation.

## Methods

### Ethics Statement

The study involved chart review, did not include any direct patient involvement, and the data was analyzed anonymously. The study was performed with the approval of the IRBs of the three participating institutions: University of California, Davis Medical Center, Sacramento, California, USA; Children's Hospital Boston, Boston, Massachusetts, USA; Children's Hospital & Research Center Oakland, Oakland, California, USA.

### Subjects and data

Clinical observations of African American and Hispanic American males and females made at presentation (first visit after determination of hyperglycemia) were obtained from medical records. From 285 charts of African American patients, 58 patients were excluded due to missing data from presentation (n = 50) or unclear diagnosis (n = 8). Data were abstracted from the remaining 227 medical records; 224 of these patients were between the ages of 2 and 18 and were used in the model (Table 1). From 168 charts of Hispanic American patients, 56 patients were excluded due to missing data from presentation (n = 21), unclear diagnosis (n = 28), and diagnosis of diabetes other than T1D or T2D (n = 7). Data were abstracted from the remaining 112 medical records; 105 of these patients, between the ages of 2 and 18, were used for the model (Table 1). Data from children younger than 2 years of age were not used in any analysis that included BMI z-score because BMI measurement in children under 2 may be inaccurate. Presentation dates in the data set spanned two decades, beginning in the 1980s.

**Table 1:** Characteristics of Patients in the Age Range of 2–18 years.

	African American				Hispanic American			
	T1D	N	T2D	N	T1D	N	T2D	N
Number of patients	124		100		69		39	
**Gender**								
% female	43%		72%*		51%		41%	
**BMI z-score** † mean (±sd)								
females	−0.13 (2.12)	53	2.11 (0.70)	72	0.36 (1.80)	35	2.34 (0.34)	16
males	0.03 (2.50)	71	2.43 (0.47)	28	0.43 (1.28)	34	2.31 (0.35)	23
**% z-score ≥2** ‡								
males plus females	16		79		12		87	
**Age at presentation** ** mean (± sd)								
females	7.4 (3.8)	53	13.3• (2.5)	72	8.8 (3.6)	35	11.7 (2.4)	16
males	9.4 (4.1)	71	14.4 (1.8)	28	8.1 (3.8)	34	13.7 (1.7)	23
**C-peptide** § (nmol/L)								
mean (± sd)	0.47 (0.33)	22	3.18 (2.08)	43	1.08 (1.76)	17	3.51 (2.04)	14
high C-peptide	6%	16	93%	30	9%	11	100%	4
**GAD65 antibodies**								
% positive	61%	59	0%	47	56%	55	13%	32

* proportion of females in T2D vs.T1D, p<0.0001.

† mean BMI z-score higher in T2D than in T1D in both genders and in both ethnicities, p<0.0004 for all comparisons tested.

‡ % of patients of both genders.

** males significantly older than females in African American T1D (p = 0.012), African American T2D (p = 0.037), Hispanic American T2D (p = 0.0091).

• African American T2D females older than Hispanic American T2D females, p<0.03.

§ For C-peptide, normal is less than 1.36 nmol/L.

The patients were seen at three medical centers: Children's Hospital and Research Center Oakland, Oakland, California, USA; UC Davis Medical Center, Sacramento, California, USA and Children's Hospital Boston, Boston, Massachusetts, USA. The study involved chart review and did not include any direct patient involvement. The study was performed with the approval of the Institutional Review Boards of the three participating institutions. Racial/ethnic status was abstracted from chart data and confirmed by clinician interview.

Data from patients with questionable race/ethnicity, borderline diabetes (e.g., impaired glucose tolerance), diabetes secondary to another condition or treatment (e.g., anti-inflammatory steroid treatment), or known diagnosis of maturity onset diabetes in youth (MODY) were excluded. Because these data were collected retrospectively, with some records many years old, the date of diagnosis and the record of height and weight did not always coincide exactly. Records were used for the study only when height and weight measurements were recorded within one month of presentation. Results of HbA1c analysis were only used if the measurement was made within one month of presentation. Family history of diabetes and presence of acanthosis nigricans (an indicator of high blood insulin levels in type 2 diabetes patients) were considered for use as additional variables in the logistic regression analyses, but data were too sparse to allow their inclusion. Patients younger than two years of age at presentation were excluded from analyses involving BMI z-score.

Established, physician-confirmed diabetes diagnoses were those recorded after at least one year of treatment. The physician used his or her clinical judgment, paired with either direct knowledge of the patient and clinical laboratory data, or thorough examination of both the recorded observational data and the clinical laboratory data to establish the diagnosis. Observational data used in diagnosis included gender, age, height, weight, family history, polyuria, and polydipsia. Clinical laboratory data included some combination of: levels of autoantibodies known to be associated with beta cell destruction (ICA, IAA, GAD65, and IA-2), blood glucose, C-peptide, glycated hemoglobin (HbA1c), blood pH, and diabetes medication. At the time of data abstraction, African American patients in the study had been treated for a mean of 4.9 years (range 1 to 18 years), and Hispanic American patients had been treated for a mean of 3.7 years (range 1 to 10 years). The respective median treatment periods were 4 and 3.5 years. Data from both genders were combined: (1) for determination by race/ethnicity of T2D and T1D rates of decline in age at presentation over the last two decades; (2) for determination by race/ethnicity of mean glycated hemoglobin values in T1D and T2D patients at presentation; and (3) for determination of changes in BMI z-score vs. patient age at presentation.

The logistic regression analysis of three variables – age at presentation, BMI z-score at presentation, and gender – were used to determine sensitivity (correct prediction of T2D), specificity (correct prediction of T1D), and the odds ratios of each variable for risk of T2D.

### Statistical Analysis

Graphical representations, statistical significance of differences between data groups, and bivariate linear regression analyses were generated with GraphPad Prism 5 (GraphPad Software, Inc., La Jolla, CA, USA). Pearson's chi-square test with one degree of freedom was used to test for a statistically significant difference in the subgroup distribution of females and males in each type of diabetes. Group means were compared for statistically significant differences by an unpaired t-test with Welch's correction, applied when equal variances cannot be assumed. Statistical differences between slopes of bivariate linear regression lines were determined by an F test.

Multivariate logistic regression analysis was performed with SAS version 9.2 (Cary, North Carolina, USA). From the pool of 224 African American patients, aged 2–18, 150 were randomly selected for the initial analysis. The remaining 74 African American patients served as a replication cohort. From the Hispanic American patient data set, 105 patients, aged 2–18, were submitted for multivariate logistic regression analysis. The statistician was blinded to the patients' diagnoses by physicians until after the computations were complete.

Area under the receiver operating characteristic curves (AUC of ROC curves) was calculated in GraphPad Prism 5 by entering 1) percent sensitivity and 2) 100% minus percent specificity directly into the GraphPad Prism 5 table. The GraphPad program was used only to calculate AUC, not to compute sensitivity and specificity; those values were taken directly from SAS multivariate logistic regression analysis. For each probability computed by SAS, the associated percent specificity was subtracted from 100% to calculate the false positives values. These were entered into GraphPad to create the ROC graph and compute the AUC.

## Results

### Gender distribution

Gender distribution differed significantly (p<0.0001) between the African American T2D patients (72% female) and the African American T1D patients (43% female). However, no significant difference was observed between T2D and T1D gender distribution in the Hispanic American patients (p = 0.42) (Table 1).

### BMI z-score

BMI is a gender-specific measure of body mass index (BMI) that is normalized for age. The BMI z-score for a given BMI is the number of standard deviations away from the BMI norm for gender and age. For T1D, BMI z-scores were highly variable in both African American and Hispanic American patients, as shown by the large standard deviations of the group means (Table 1). T2D BMI z-scores were not only much higher than T1D scores but also less variable.

Obesity was common among T2D patients at presentation. In addition, a significant percentage of T1D patients were obese (BMI z-score ≥2) at this time. The percentage of obese T1D African American patients was 16% and in Hispanic American patients was 12% (Table 1).

**Figure 1:**
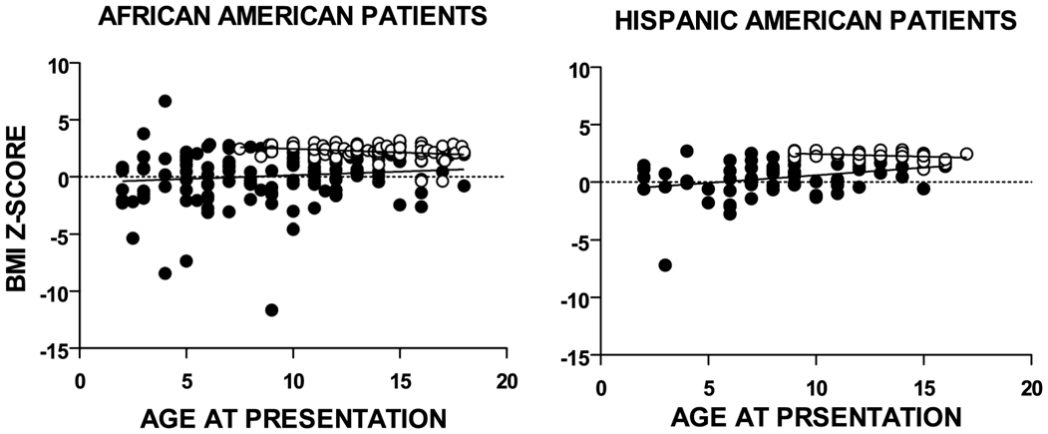
Linear regression of BMI z-score vs. age at presentation. Age range at presentation, age 2–18. The T1D BMI z-score tended to increase as the age at presentation increased from 2 to 18 years. In African American patients the rate of increase was not significant, however the rate of the increase in T1D BMI z-score of Hispanic American patients was small but significant (p = 0.008). In T2D patients, the trend of BMI z-score was down as the age of presentation went up. The rate of decrease in African American patients was small but significant (p = 0.01); in Hispanic American patients the rate of decrease was insignificant. (•) T1D, (o) T2D.

BMI z-scores increased slightly but steadily with an increase in the age at presentation in T1D patients and decreased slightly but steadily in T2D patients (Figure 1). Thus, the T2D scores tended to slightly diminish toward the norm, but were still very high over the onset age range of 2–18 years.

### Age at presentation

Mean age at onset was lower for females than for males (p<0.04), with the exception of T1D Hispanic American females whose mean age was older than that of T1D males (Table 1).

**Figure 2:**
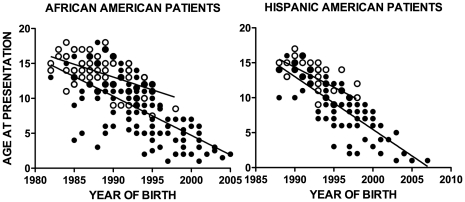
Linear regression of age at presentation vs. year of birth. Age range at presentation, age 1–18. African American T1D regression line slope was −0.56 (r^2^ = 0.46); T2D slope was −0.37 (r^2^ = 0.32). Hispanic American T1D regression line slope was −0.76 (r^2^ = 0.66); T2D slope was −0.57 (r^2^ = 0.44). The African American T1D and T2D rates of decrease in age were significantly different (p = 0.04). The Hispanic American T1D and T2D rates of age decrease were not significantly different. (•) T1D, (o) T2D.

**Figure 3:**
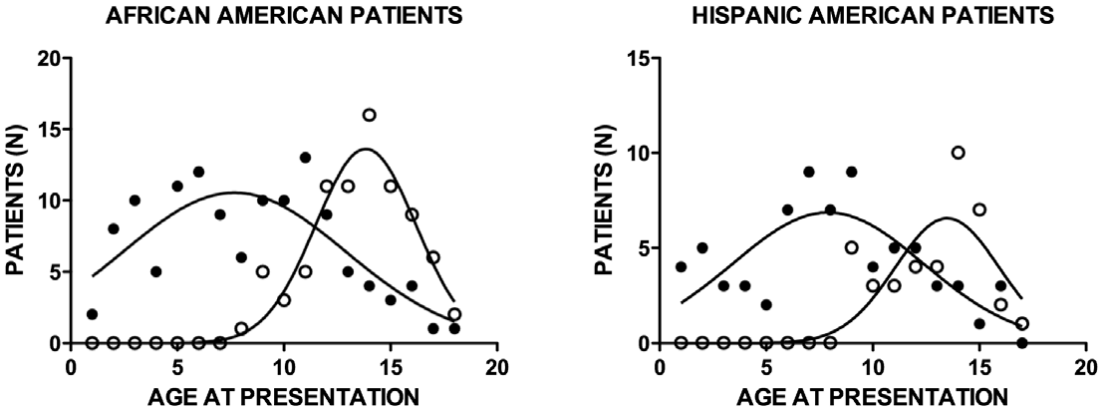
Number of patients per age at presentation. Age at presentation of T2D began to overlap age at presentation of T2D at age 8 or 9. (•) T1D, (o) T2D.

The decline in age at presentation for T1D and T2D has been shown in other studies [1], [11]. The study reported here demonstrates similar decreases in age at presentation (Figure 2). The decreases in age at presentation illustrate a confounding factor in the differential diagnosis of T2D and T1D and pose a challenge to developing a diagnostic aid for use at presentation. The overlapping of age at presentation between T1D and T2D patients is illustrated graphically in Figure 3. The overlap was particularly pronounced in the age range of 10 to 15 years.

Combining the data from both minority groups, most of the younger T2D patients were females. Of the 51 male T2D patients in the study only one presented younger than 10 years of age. In contrast, 14% of T2D females (12 out of a total of 88 AA and HA females) were under 10 years of age at presentation. This difference was statistically significant (p = 0.031).

### Glycated hemoglobin (HbA1c) at onset of diabetes

The HbA1c data recorded within one month of presentation were available for a subset of 97 African American patients and a subset of 95 Hispanic American patients. No gender differences were observed in HbA1c for either AA or HA patients (data not shown). The mean HbA1c in T2D Hispanic American patients was significantly less than that in T1D (p<0.002) (Table 2). Mean glycated hemoglobin in African American patients was similar in T2D and T1D.

**Table 2:** Glycated hemoglobin at onset of diabetes.

	T1D HbA1c	N	T2D HbA1c	N	p-value*
**African American**					
mean (± sem)	10.78 (±0.31)	52	10.96 (±0.45)	45	0.74
**Hispanic American**					
mean (± sem)	11.34 (±0.31)	59	9.41 (±0.44)	36	<0.002

* mean T1D vs. mean T2D.

### Multivariate logistic regression and prediction of T2D

In the abstracted data, several variables differed significantly between T1D and T2D patients, including BMI, gender, C-peptide, anti-GAD autoantibodies, current use of oral diabetes medications, hyperpigmentation, and family history for T2D (data not shown). Of the subset of variables that can be assessed immediately upon patient presentation, only age, gender, and BMI z-score had sufficient data available for use in constructing a predictive model. One hundred fifty of 224 eligible African American patient records were randomly selected for inclusion in the analysis constructing the model. Using a cut point of 0.5, results of regression analysis gave a sensitivity of 92% (correct prediction of T2D) and specificity (correct prediction of T1D) of 89%. Application of the model from this logistic regression analysis to the remaining 74 African American patient records validated the results: sensitivity was 91% and the specificity was 93%.

Application of the model generated from African American data to a cohort of 89 Hispanic American patients produced lower sensitivity and specificity values: 79% and 87%, respectively. The Hispanic American data set was then expanded until the number of eligible patients reached 105. With 105 Hispanic American patients, logistic regression produced a model with sensitivity and specificity of 92% and 90%, respectively. The progress of the model development is summarized in Table 3.

**Table 3:** Logistic Regression Model Development in African American (AA) and Hispanic American (HA) Patient Groups.

Model (M)	Tested on:	No. patients	Sensitivity	Specificity
**M-AA created**	----	150	92	89
**M-AA test** *	AA	74	91	93
**M-AA test** **	HA	89	79	87
**M-HA created**	----	105	92	90

* The model created with AA data was tested in a separate cohort of AA patients.

** The model created with AA data was tested in HA patients.

The odds ratios obtained illustrated the importance of female gender as a risk factor for T2D in African Americans and showed that BMI was prominent as a risk factor for both African American and Hispanic Americans. Increasing age at presentation increased the odds of T2D in both African American and Hispanic American patients (Table 4).

**Table 4:** T2D Odd Ratios (95% confidence limits).

	African American	Hispanic American
Variable	Odds Ratio (95%CL)	Odds Ratio (95%CL)
Female gender	12.95 (3.54 to 47.4)	0.74 (0.19 to 3.02)
BMI z-score	4.10 (2.19 to 7.68)	25.6 (4.85 to 135.0)
Age at presentation	1.97 (1.52 to 2.53)	1.49 (1.16 to 1.93)

### ROC (receiver operating characteristic) curves

**Figure 4:**
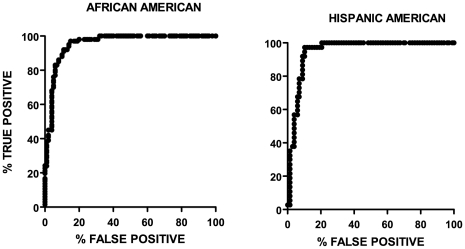
ROC curves. The African American data set contained data from 150 patients and the Hispanic American data set contained data from 105 patients.

The ROC curve is a measure of the utility of the test for prediction of T2D. A ROC area under the curve (AUC) ranges from 0.5 (no predictive value) to 1.0 (perfect predictive value). The AUC of 0.855 (95% CI 0.814–0.896) in the African American patient group indicates that this test for T2D is very useful in differentiating between T2D and T1D. The Hispanic American patient group data yielded a similarly good result: AUC of 0.858 (95% CI 0.808–0.908). The ROC curves are shown in Figure 4.

## Discussion

Thirty years ago, T2D was rare in children. Generally, children presenting with elevated blood glucose were considered T1D patients and adults presenting with elevated blood glucose were considered T2D patients. In the interim, previously unrecognized forms of diabetes, including MODY (maturity onset of diabetes in youth) and LADA (latent autoimmune diabetes in adults), have blurred the differential diagnosis of diabetes to some extent. The biggest contributor to difficulty in the differential diagnosis of T1D vs. T2D, however, is decreasing age of onset for both T1D and T2D, but particularly for T2D. As a consequence of the decreasing age at presentation over the last two decades, the age at presentation of T2D overlapped that of T1D in both African American and Hispanic American patients. Decreasing age of T1D onset in our data is similar to that of several long-term studies in England, Finland, and Italy [12], [13], [14]. A dramatic decrease in the age of onset for T2D has been noted in several studies and is particularly striking in non-Caucasian groups, such as African Americans, Hispanics, and Native Americans, who are, for reasons not yet fully understood, particularly susceptible to T2D [15], [16], [17]. This makes distinction of T1D vs. T2D particularly challenging in these groups and is the basis for the current study.

Overweight/obesity was a strong predictor of T2D in both African American and Hispanic American youth; however, the odds ratio for BMI z-score was far greater in HA (25.6) than in AA (4.1). Many lines of evidence support the notion that race/ethnicity can be an important factor in T2D susceptibility. Prevalence of obesity varies among ethnic groups. In a recent study, prevalence of obesity among 4 year old U.S. children was reported as: 31% in American Indian/Native Alaskan, 22% in Hispanic American, 21% in African American, 16% in European American, and 13% in Asian American [18]. Among youth with T2D, Asians had a lower mean BMI than did Pacific Islanders, 33.7 vs. 42.4, suggesting that Asian youth may be at risk for T2D at a lower BMI than other racial/ethnic groups [19]. Similarly, a study of adults found that South Asian T2D patients had lower BMI than did European white T2D patients [20]. Thus, although BMI is strongly correlated with T2D, other factors, perhaps genetic or cultural, combine with BMI to determine susceptibility. Our data show that the predictive test we are developing performs much better when used for patients who belong to the group with which it was created, i.e., the predictive value for HA patients is better when the HA-specific model is used, rather than the AA-specific model. Taken together, all of these observations underscore the fact that race/ethnicity plays a role in T2D susceptibility and should be taken into account when attempting to distinguish diabetes type.

The uneven gender distribution in African American T2D reported here, with significantly more females than males, is consistent with other studies [2], [7]. The absence of this gender bias in Hispanic American patients is also consistent with previous reports [2], [7]. The difference in gender distribution between T1D and T2D in African American but not in Hispanic American patients, the lesser prominence of BMI in risk of T2D in African American patients, and the difference in T1D and T2D glycated hemoglobin in Hispanic American patients at onset may all reflect differences in underlying genetic influences in the two populations in this study.

Other research supports the notion of race-specific genetic differences that could affect T2D risk. Examination of healthy pediatric subjects showed higher insulin secretion and lower insulin clearance in African American subjects compared to European American subjects [21], [22]. The metabolic syndrome, which can precede type 2 diabetes, has different characteristics in European Americans, African Americans, and Hispanic Americans [23]. Fatty acid metabolism is different in African Americans compared to European Americans due to a genetic difference in a key enzyme [24].

This study demonstrates that only a few variables recorded at presentation may provide a model that could aid in differential diagnosis of T1D and T2D in African American and Hispanic American pediatric patients at the time of presentation. This study also provides evidence that models to predict T2D from data recorded at presentation should be race/ethnicity specific because the relative risk of T2D as indicated by gender and BMI z-score is different in African American and Hispanic American patients. These results suggest that other racial/ethnic groups may have their own levels of risk for T2D due to age, gender, and BMI z-score.

A larger study, with data collected prospectively, is currently in the planning stage. Data from this survey-type study will be utilized to replicate the present work, improve the accuracy of prediction of T2D, and test whether the addition of more observational variables, such as presence or absence of acanthosis nigricans, can further improve the prediction of diabetes type. The data presented here suggest that an immediate, simple, rapid, and inexpensive test can be developed to predict diabetes type at presentation in underserved minority children and youth.
